# Investigating the effects of Pirfenidone on TGF-β1 stimulated non-SMAD signaling pathways in Dupuytren’s disease -derived fibroblasts

**DOI:** 10.1186/s12891-019-2486-3

**Published:** 2019-03-30

**Authors:** Chaoming Zhou, Yael Zeldin, Mark E. Baratz, Sandeep Kathju, Latha Satish

**Affiliations:** 1 Department of Plastic Surgery, University of Pittsburgh, Pittsburgh, PA 15261 USA; 2 McGowan Institute for Regenerative Medicine, Pittsburgh, PA 15219 USA; 3 Shriners Hospitals for Children-Cincinnati, Cincinnati, OH 45229 USA; 4 Department of Orthopaedic Surgery, University of Pittsburgh, Pittsburgh, PA 15261 USA; 5 Department of Pathology and Laboratory Medicine, University of Cincinnati, 3229 Burnet Avenue, Cincinnati, OH 45229 USA; 6 Lumix Biomedical and Surgical Consulting, Pittsburgh, PA USA

**Keywords:** Dupuytren’s contracture, Palmar fascia fibrosis, Carpal tunnel, AKT, ERK1/2, p38MAPK and myosin light chain (MLC)

## Abstract

**Background:**

Dupuytren’s disease (DD) is a progressive, debilitating condition of the hand that can eventually cause contractures of the affected fingers. Transforming growth factor- β1 (TGF-β1) has been reported to play a key role in DD pathology. Increased expression of TGF-β1 has shown to be the main stimulator of myofibroblast activity and in DD contractures. Pirfenidone (PFD), a small active molecule possess the ability to inhibit TGF-β1-mediated action in various fibrotic disorders. Our recent published findings show that PFD reduced TGF-β1-mediated cellular functions implicated in DD through SMAD signaling pathways. In the present study, the effect of PFD on TGF-β1-mediated non-SMAD signaling pathways were investigated in both carpal tunnel (CT) - and DD-derived fibroblasts.

**Methods:**

Fibroblasts harvested from Dupuytren’s disease (DD) and carpal tunnel (CT) tissues were cultured in the presence or absence of TGF-β1 (10 ng/ml) and/or PFD (800 μg/ml). Cell lysates were analyzed using Western blots. Equal amounts of proteins were loaded to determine the phosphorylation levels of phosphatidylinositol-3 kinase (PI3K/AKT), extracellular regulated kinases (ERK1/2), p38 mitogen-activated protein kinase and Rho family related myosin light chain (MLC).

**Results:**

We show that the TGF-β1-induced phosphorylation of AKT was significantly decreased by the addition of PFD (800 μg/mL) in both CT- and DD-derived fibroblasts. Interestingly, there was no significant difference in the phosphorylation levels of both ERK and p38 on TGF-β1- induced cells in both CT-and DD-derived fibroblasts. But, PFD significantly decreased the TGF- β1-induced phosphorylation levels of ERK1/2 in both CT- and DD- cells. In contrast, PFD significantly decreased the basal and TGF- β1-induced phosphorylation levels of p38 in DD-derived fibroblasts. TGF- β1-induced phosphorylation levels of MLC was decreased by PFD in DD-derived fibroblasts.

**Conclusions:**

These in-vitro results indicate for the first time that PFD has the potential to inhibit TGF-β1-induced non-SMAD signaling pathways in both CT- and DD-derived fibroblasts but pronounced statistically significant inhibition on all molecules was observed only in DD-derived fibroblasts. Our previous studies show that PFD can inhibit TGF-β1- induced SMAD signaling pathway proteins, namely p- SMAD2/SMAD3. These broad and complementary actions suggest PFD as a promising candidate to inhibit the TGF-β1- mediated molecular mechanisms leading to DD fibrosis.

## Background

Transforming growth factor-beta 1 **(**TGF-β1) is widely implicated in fibrotic diseases including Dupuytren’s disease (DD; also known as palmar fascia fibrosis). DD is the most common heritable fibroproliferative disorder of the palmar fascia of the hand. It affects 5–25% of people of European descent, and there is evidence that the prevalence of DD is increasing [1, 2]. There is a strong genetic predisposition to DD, and there is a strong indication that DD has a substantial hereditary component [3]. Similarly, there is evidence that multiple non-genetic factors, such as smoking, alcohol intake, diabetes, and hyperlipidemia, also play a role in disease development [4]. In DD pathogenesis, subcutaneous fat is progressively replaced by a relatively avascular fibrous tissue, which develops as nodules in the palm and extends distally into the digits as thickened cords [5], which draw the fingers into a flexed posture. The options for treating DD include the percutaneous release of the offending cords [6], injection of collagenase followed 1–2 days later with manipulation to rupture the cords [7] and surgical resection of the cords [8]. All of these treatment strategies have a high risk of recurrence [9].

While the molecular and cellular triggers remain difficult to elucidate during DD development, the progressive increase in extracellular matrix protein and subsequent contraction clearly suggests a role for myofibroblasts. Myofibroblasts have the ability to synthesize collagen and other extracellular matrix components in the palmar fascia leading to digital contractures [10, 11]. A number of studies indicate that growth factors control the growth and proliferation of myofibroblasts. A role for several cytokines including FGF, IL-1, TGF-β1, and PDGF, and all have been shown to influence DD pathogenesis [12, 13]. TGF-β1, a well-known pro-fibrotic growth factor, is up-regulated in DD- tissue [14, 15] and is a major factor in the transformation of fibroblasts to myofibroblasts in DD [16–18]. Previous studies have described the activation of fibroblasts by TGF- β and other cytokines as a major mechanism driving the fibrotic processes in DD and other fibroses [19]. TGF-β_1_ stimulation leads to increased contractile force in DD-cells [20–22] and also leads to up-regulation of key ECM components, such as fibronectin and type I collagen [16].

TGF-β1 signaling can occur through both canonical and non-canonical pathways. In canonical TGF- β1 signaling, binding of TGF-β1 to its receptors activates SMAD 2/3-dependent signaling; activated SMAD 2/3 complexes with SMAD 4 and translocates into the nucleus and regulates the expression of TGF-β-responsive genes [23]. The TGF-β-induced non-canonical, SMAD-independent pathways include various branches of MAP kinase (MAPK) pathways, Rho-like GTPase signaling pathways, and phosphatidylinositol-3-kinase (PI3K)/AKT pathways [24]. TGF-β1’s pleiotropic effects on DD cells, especially fibroblasts, make it an ideal candidate to target in the fight against Dupuytren’s fibrosis.

Pirfenidone (PFD; 5-methyl-1-phenyl-2 (1H)-pyridone) is currently used to treat patients with Idiopathic Pulmonary Fibrosis, a condition with similarities to Dupuytren’s Disease [25]. We have shown that PFD inhibits TGF-β1-induced transformation of fibroblasts to myofibroblasts and inhibits ECM production, mainly type I/type III-collagens and fibronectin in DD-derived fibroblasts. PFD also inhibits TGF-β1-induced cell migration, proliferation and contractile ability in DD-derived fibroblasts. We further showed that TGF-β1-induced phosphorylation of SMAD 2/SMAD 3, a key factor in the TGF-β1 signaling pathway, was attenuated by the addition of PFD [21]. In the present study, we investigated the action of PFD on TGF-β_1_-induced non-SMAD signaling pathways in DD-derived fibroblasts as both SMAD-dependent and- independent signaling pathways elicited by TGF-β1 have a significant role in mediating fibrosis. Our focus was to examine the changes that PFD could elicit in the basal- and TGF-β1-induced phosphorylation levels of extracellular signal-regulated kinase 1/2 (ERK1/2); Akt, a serine/threonine-specific protein kinase; p38 mitogen-activated protein kinases and myosin light chain (MLC), a subunit of myosin.

## Methods

### Cell culture

Patients undergoing surgery for resecting DD- cord and CT- facial tissues were consented using an IRB approved protocol conforming to the ethical guidelines of the 1975 Declaration of Helsinki. The discarded tissues were collected, and primary cultures of fibroblasts were established as described previously [16]. Fibroblasts were cultured in the α-MEM (Invitrogen™, ThermoFisher Scientific, Pittsburgh, PA) containing 10% FBS and 10% penicillin/streptomycin (Gibco®, ThermoFisher Scientific) and maintained in an incubator with 5% CO_2_. Cells were used between passages 2 to 7 for all of the experiments.

### Western blotting

Fibroblasts harvested from DD- cord and CT- tissues (2 × 10^5^) were cultured for 24 h in 6 well plates with α-MEM containing 10% FBS and penicillin/streptomycin antibiotics. The medium was then changed to α-MEM supplemented with 0.1% dialyzed FBS for an additional 24 h following which the cells were treated with PFD (Toronto Research Chemicals, Ontario, Canada; 800 μg/mL) with or without TGF-β1 (Peprotech, Rocky Hill, NJ; 10 ng/ml) at indicated times (5 mins for p38, 15 mins for ERK1/2 and 24 h for both AKT and MLC). After treatment, the cells were washed with cold PBS and lysed with M-PER™ Mammalian Protein Extraction Reagent (ThermoFisher Scientific) with added proteinase inhibitors (cOmplete ULTRA Tablets, Mini, EDTA-free), and Phosphostop purchased from Roche Diagnostics Corporation (Indianapolis, IN). Protein samples between 15 and 30 μg were resolved by SDS-PAGE using standard methods. Western blotting procedures were followed using the primary antibodies purchased from Cell Signaling Technology (Danvers, MA). After blocking with 5% BSA, proteins were probed with phospho-p44/42 MAPK (Erk1/2) (Thr202/Tyr204) (cat. no. 4370) or phospho-Akt (Ser473) (D9E; cat.no. 4060) at a 1:1000 dilution or phospho-myosin light chain 2 (Thr18/Ser19; cat.no. 3674) or phospho-p38 MAPK (Thr180/Tyr182) (28B10; cat.no. 9216) at 1:500 dilution. GAPDH (Novus Biologicals, Littleton, CO; cat.no. NB-300-221) was used as a loading control at 1: 2500 dilution. After incubating with primary antibody overnight at 4 °C, the membranes were washed and incubated for 1 h at room temperature with IRDye 800CW Gt Anti-Rabbit IgG (H + L) antibody and IRDye 680LT Anti-Mouse IgG (H + L) antibody (1:20,000) (Li-COR Bioscience, Lincoln, NE). Infrared fluorescence was detected using Odyssey® Imaging System (Li-COR Bioscience). The protein bands were analyzed using NIH Image J 1.44p. The phospho- protein was normalized against the corresponding total protein.

### Statistical analysis

Experimental values are expressed as means ± standard error mean (SEM) of three independent studies from each of CT- and DD- derived fibroblasts. GraphPad Prism version 8 was used to determine the statistically significant differences between various treatments. Data analysis was performed using One way ANOVA (and nonparametric or mix), by choosing both Sidak and Dunnett’s multiple post-hoc comparisons. A *p* value < 0.05 was considered statistically significant comparing the treatments within CT- and DD-cells.

## Results

### PFD inhibition of TGF-β1-induced phosphorylation of PI3K/Akt in CT- and DD-derived fibroblasts

**Fig. 1:**
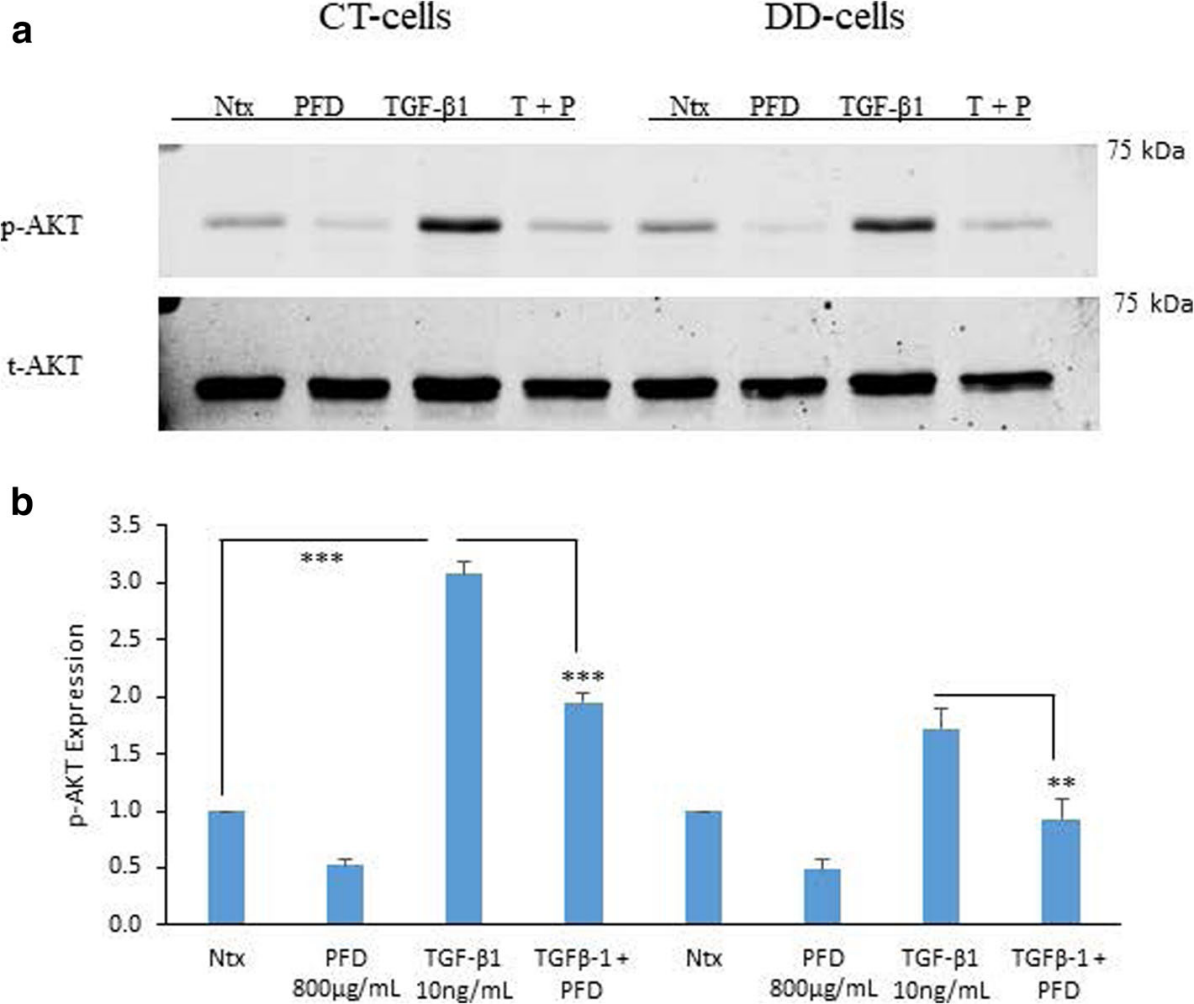
Pirfenidone significantly reduced TGF-β1-induced phosphorylation of Akt in CT- and DD-derived fibroblasts. **a** CT- and DD-cord derived fibroblasts derived from three different patient samples (*N* = 3/group) were maintained in α-MEM medium containing 0.1% dialyzed FBS for 24 h. After 24 h, cells were either left as controls or treated with PFD (800 μg/ml) in the presence or absence of TGF-β1 (10 ng/ml) for an additional 24 h. Cell lysates were subjected to Western blot analyses to determine the expression of phosphorylated Akt. **b** Densitometry results are reported as the ratio of phosphorylated Akt protein level to GAPDH expression. Values are means ± standard error mean (SEM) of three independent studies from each of CT- and DD- derived fibroblasts. **b** Shown here is a representative image of Western blots from three different cultures of CT- and DD-cord derived fibroblasts, each showing similar results. ***p < 0.0001, **p < 0.0059. Ntx; No treatment, PFD- Pirfenidone, TGF-β1, T + P; TGF- β1 + Pirfenidone

The PI3K/Akt pathway is an intracellular signaling pathway activated by various growth factors and cytokines such as TGF-β1. The phosphorylation levels of AKT increased when stimulated with TGF- β1- in both CT- and DD-cells but statistically significant increase was seen only in CT cells (*p* < 0.0001). Addition of PFD reduced TGF-β1-induced phosphorylation of Akt (from 3.08 ± 0.1 to 1.95 ± 0.09 for CT; p < 0.0001 and from 1.78 ± 0.2 to 0.82 ± 0.5; *p* < 0.0059 for DD) (Fig. 1a and b).

### PFD inhibition of both ERK1/2 and p38 phosphorylation levels in both CT- and DD-derived fibroblasts

**Fig. 2:**
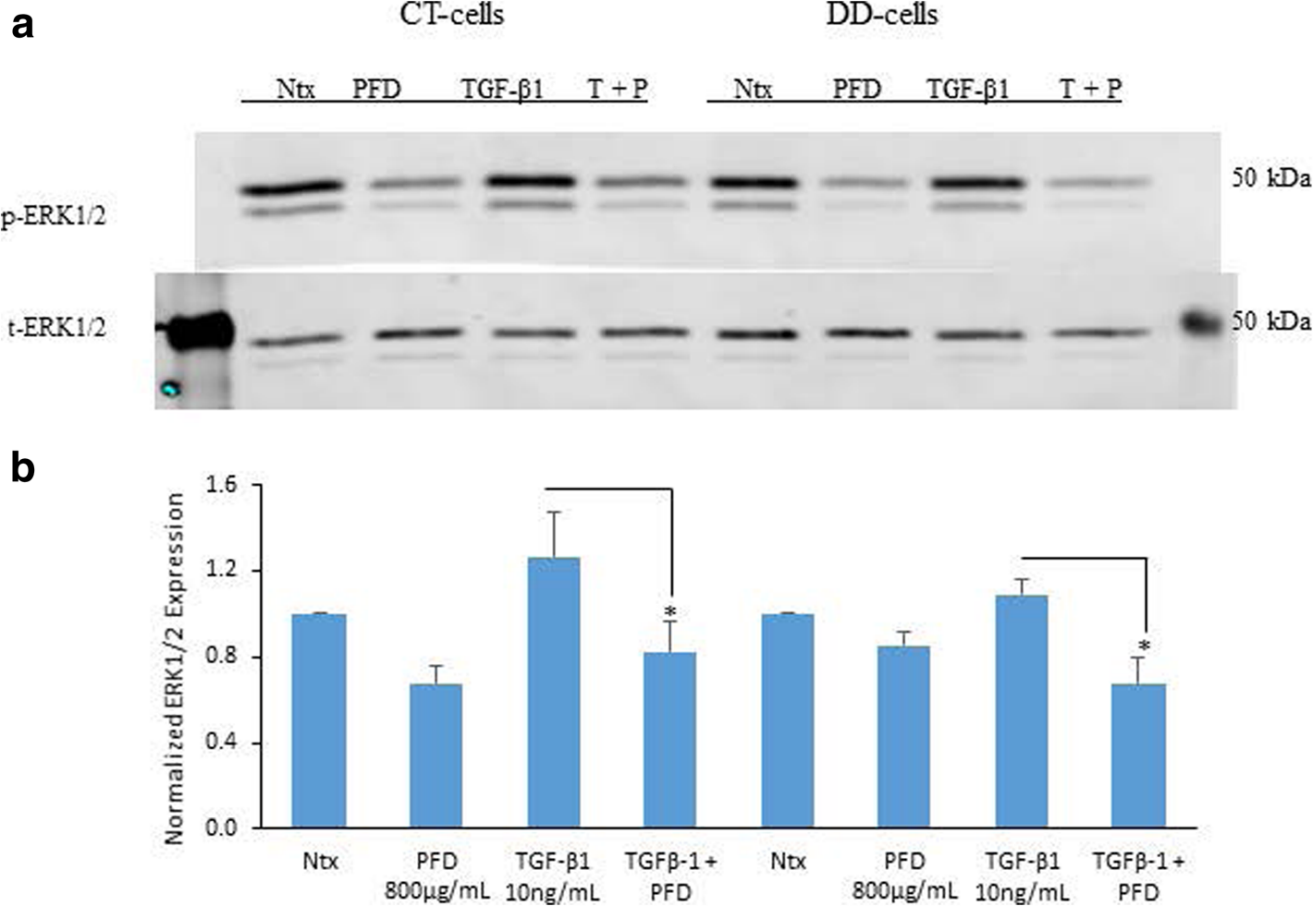
Pirfenidone significantly reduced TGF-β1-stimulated phosphorylation of ERK1/2 in both CT- and DD-derived fibroblasts. **a** CT- and DD-cord derived fibroblasts derived from three different patient samples (*N* = 3/group) were maintained in α-MEM medium containing 0.1% dialyzed FBS for 24 h. After 24 h, cells were either left as controls or treated with PFD (800 μg/ml) in the presence or absence of TGF-β1 (10 ng/ml) for 15 min. Cell lysates were subjected to Western blot analyses to determine the expression of phosphorylated ERK1/2. **b** Densitometry results are reported as the ratio of phosphorylated ERK1/2 protein level to GAPDH expression. Values are means ± standard error mean (SEM) of three independent studies from each of CT- and DD- derived fibroblasts. Shown here is a representative image of Western blots from three different cultures of CT- and DD-cord derived fibroblasts, each showing similar results. *p < 0.04, . Ntx; No treatment, PFD- Pirfenidone, TGF-β1, T + P; TGF- β1 + Pirfenidone

MAPK-activated protein kinases are related serine/threonine kinases. They respond to mitogenic and stress stimuli through proline-directed phosphorylation and activate the kinase domain by extracellular signal-regulated kinases 1 and 2 (ERK1/2) and p38 MAPKs. We examined the phosphorylation of ERK1/2 and of p38 with and without TGF-β1 stimulation and PFD treatment in CT- and DD-derived fibroblasts. Interestingly, phosphorylation levels of both ERK1/2 and p38 MAPKs did not significantly increase when stimulated with TGF- β1 for 15 min in both CT and DD cells. Further, we found that the addition of PFD to TGF- β1-induced CT- and DD- cells significantly decreased the phosphorylation levels of ERK1/2 (*p* < 0.02) (Fig. 2a and b).

**Fig. 3:**
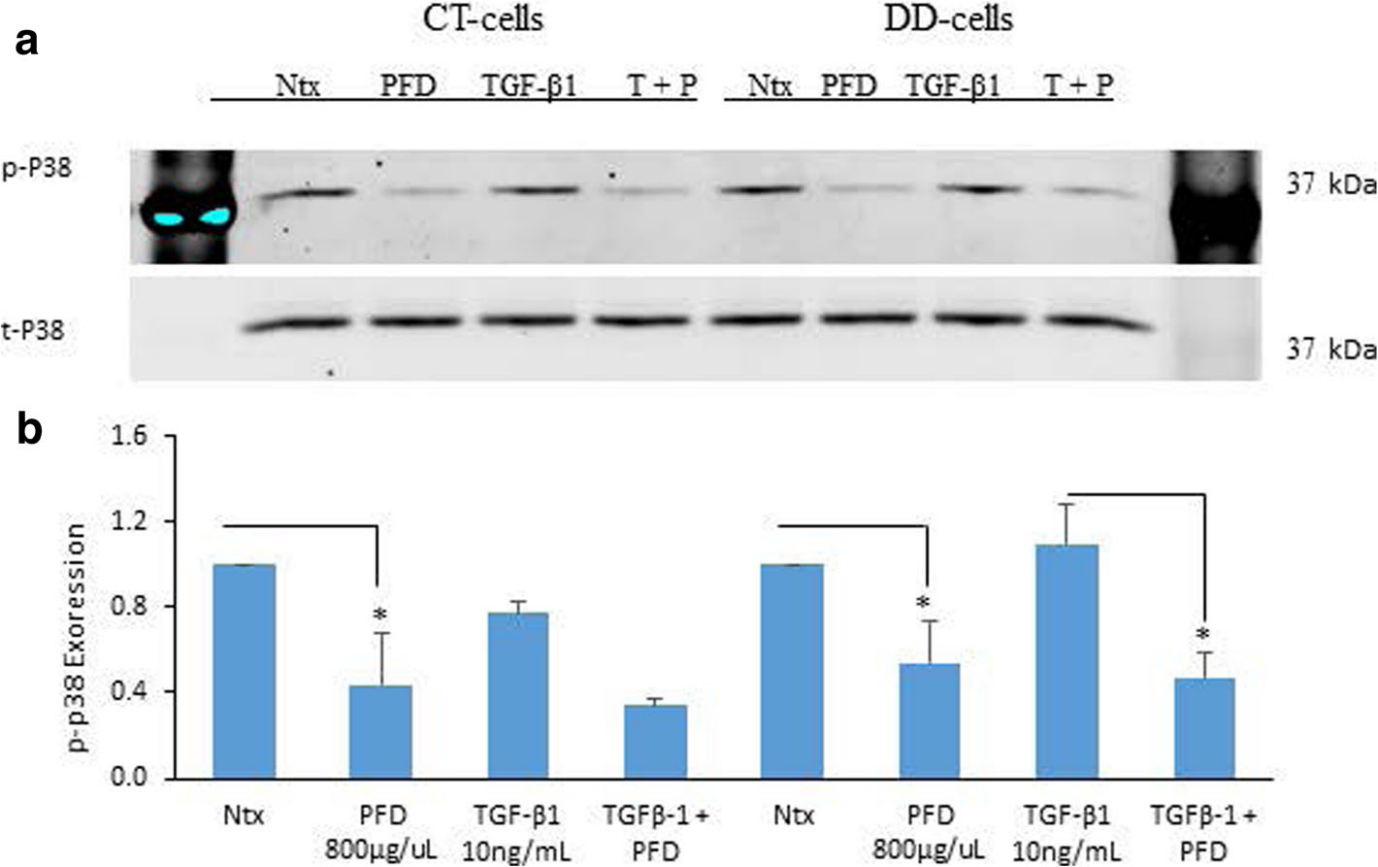
Pirfenidone reduced both TGF-β1-induced and basal phospho-p38 levels in DD-derived fibroblasts. **a** CT- and DD-cord derived fibroblasts derived from three different patient samples (*N* = 3/group) were maintained in α-MEM medium containing 0.1% dialyzed FBS for 24 h. After 24 h, cells were either left as controls or treated with PFD (800 μg/ml) in the presence or absence of TGF-β_1_ (10 ng/ml) for 5 min. Cell lysates collected were subjected to Western blot analyses to determine the expression of phospho-p38. **b** Densitometry results are reported as the ratio of phosphorylated p-38 protein level to GAPDH expression. Values are means ± standard error mean (SEM) of three independent studies from each of CT- and DD- derived fibroblasts. Shown here is a representative image of Western blots from three different cultures of CT- and DD-cord derived fibroblasts, each showing similar results. *p < 0.04. Ntx; No treatment, PFD- Pirfenidone, TGF-β_1_, T + P; TGF- β_1_ + Pirfenidone

Interestingly, our findings showed no significant stimulation in p38 phosphorylation when induced with TGF- β1 in both CT- and DD-derived fibroblasts. However, the addition of PFD decreased the phosphorylation levels of p38 in untreated (basal phosphorylation) cells of both CT- and DD-derived fibroblasts. However, PFD significantly decreased TGF-β1-induction of p38 only in DD-cells (*p* < 0.04) (Fig. 3a and b).

### PFD inhibition of TGF-β1-induced phosphorylation of myosin light chain

**Fig. 4:**
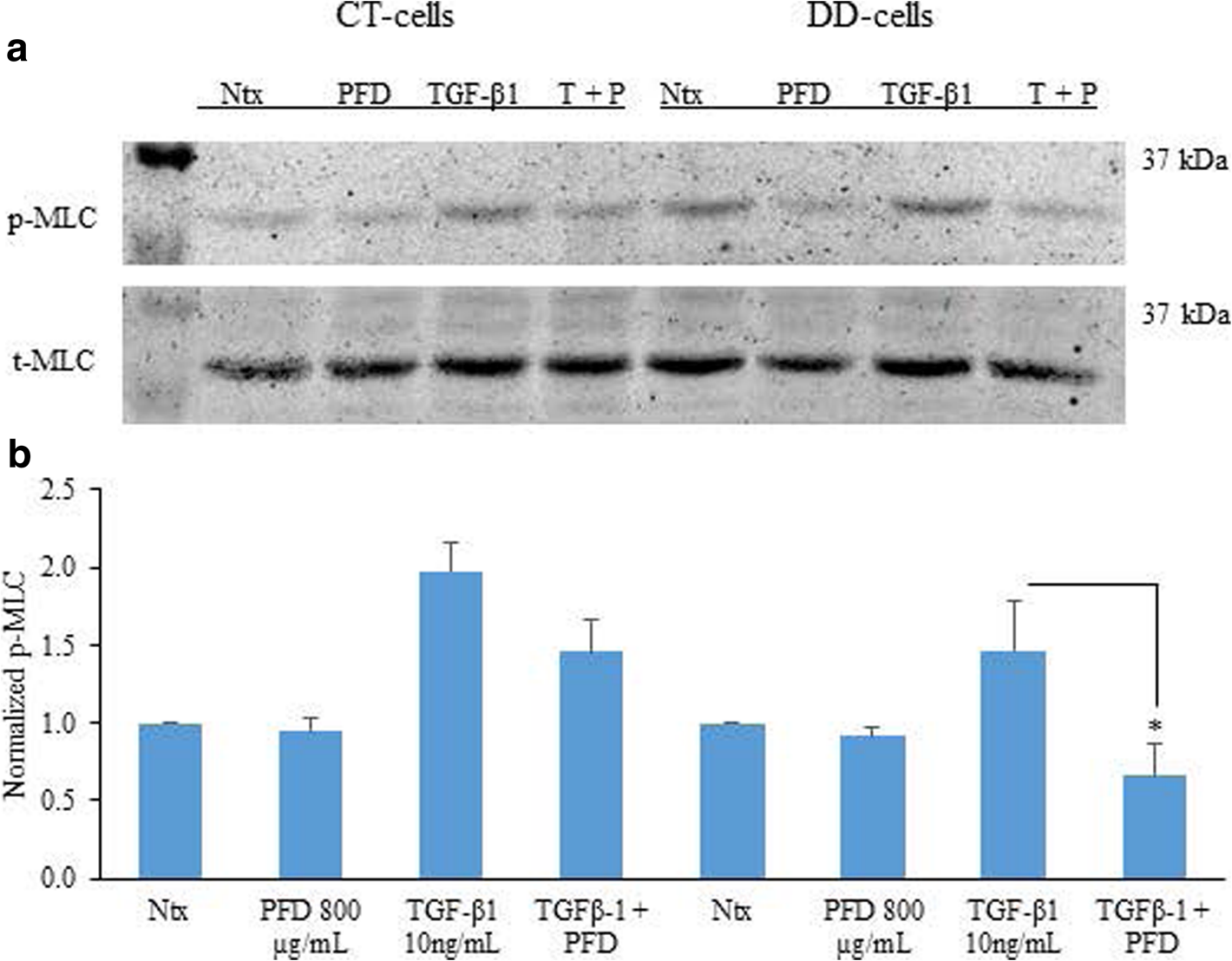
Pirfenidone significantly reduced TGF-β1-induced phosphorylation of MLC in DD-derived fibroblasts. **a** CT- and DD-cord derived fibroblasts derived from three different patient samples (*N* = 3/group) were maintained in α-MEM medium containing 0.1% dialyzed FBS for 24 h. After 24 h, cells were either treated or untreated with PFD (800 μg/ml) and in the presence or absence of TGF-β1 (10 ng/ml) for additional 24 h. Cell lysates were subjected to Western blot analyses to determine the expression of phosphorylated MLC. **b** Densitometry results are reported as the ratio of phosphorylated MLC protein level to GAPDH expression. Values are means ± standard error mean (SEM) of three independent studies from each of CT- and DD- derived fibroblasts. Shown here is a representative image of Western blots from three different cultures of CT- and DD-cord derived fibroblasts, each showing similar results. *p < 0.02,. Ntx;No treatment, PFD- Pirfenidone, TGF-β1, T + P; TGF- β1 + Pirfenidone

MLC phosphorylation plays a key role in various cellular functions including cellular morphogenesis, cell migration, and cell contraction. We found that basal phosphorylation levels of MLC were increased in DD-derived fibroblasts when compared to CT-derived fibroblasts, although this difference did not reach statistical significance. Stimulation of DD- and CT-fibroblasts with TGF-β1 for 24 h increased the phosphorylation levels of MLC and did not reach statistical significance. Interestingly, PFD did not inhibit the basal phosphorylation levels of MLC in both CT- and DD-derived fibroblasts. However, significant decrease in the TGF- β1-stimulated phosphorylation levels of MLC in DD-derived fibroblasts (*p* < 0.02) was noted (Fig. 4a and b).

## Discussion

DD is a complex disease that is characterized by the involvement of various growth factors, signaling molecules, and pathways that drive the process of fibrosis in the palm of the hands [26]. TGF-β1 has been identified as a central growth factor in activating fibroblasts to transform to myofibroblasts, resulting in excessive accumulation of ECM proteins, especially collagen. Previous biochemical and immunochemical studies have reported a decrease in type III/I collagen ratio with DD progression [27, 28]. In contrast, a study by Lam et al. (2010) quantified the different collagen types by computer analysis using stained DD tissue and documented a decrease in the amount of type III collagen as a percentage of the total collagen with the progression of the disease [29]. There is strong evidence that the blockade of TGF-β1 alone is sufficient to block experimental fibrosis in liver, kidney, lung, heart, and skin [30]. Targeting TGF- β1 may alleviate some of the adverse effects leading to fibrosis, and it may prevent the progression of DD. The small molecule PFD is an approved oral anti-fibrotic agent for patients affected with idiopathic pulmonary fibrosis [25]. PFD mainly targets TGF-β1- induced fibrosis [31]. Our previous in-vitro studies demonstrated that PFD could prevent the TGF- β_1_-induced fibroblast to myofibroblast transformation and that it inhibits ECM protein production, especially type I and type III collagen and fibronectin in DD-derived fibroblasts [21].

TGF- β1, a known pro-fibrotic growth factor elicits its effects by inducing both canonical and non-canonical signaling pathways. We have previously shown that PFD can inhibit TGF-β1- induced phosphorylation of SMAD 2/SMAD 3 in DD-derived fibroblasts, a key factor in the TGF-β1-induced canonical signaling pathway. We also reported in our previous studies that PFD inhibits TGF-β_1_-induced cell migration, cell contraction and cell proliferation in both carpal tunnel (CT) - and DD-derived fibroblasts, but that the effect was more pronounced in DD-derived fibroblasts [21]. Several studies have reported the effects of PFD on fibroblasts derived from other tissues namely, ocular, intestine, dermis and lung. In primary lung fibroblasts derived from idiopathic pulmonary fibrosis (IPF) patients authors show that combination of pirfenidone and rapamycin widen the inhibition range of ECM fibrogenic markers and prevents fibroblast migration [32] showing possibility for using combination of small molecules for anti-fibrotic treatment. Similarly, Stahnke et al. (2017) [33] show in ocular fibroblasts PFD inhibition of ECM protein deposition again suggesting PFD as a promising candidate for treating fibrosis following glucoma surgery. Sun et al. (2018) [34] present findings using intestinal fibroblasts that PFD could inhibit TGF-β1-induced SMAD and PI3K/AKT signaling pathways and suggested the application of PFD as a potential therapeutic agent for intestinal fibrosis. Another interesting finding originates from Hall et al. 2018 [35] showing evidence for PFD inhibiting TGF-β1-induced type I- and type III- collagens and targeting the p38 MAPK signaling pathway in human dermal fibroblasts which shows the capability of PFD to target dermal fibrosis.

In continuation of these findings, our interest was to determine if treatment of CT- and DD- fibroblasts with PFD could attenuate non-canonical signaling pathways induced by TGF-β1, particularly those involving pERK, pAKT, p38, and pMLC as each of these molecules have been found to play a role in cell migration, proliferation, differentiation, and contraction. Interestingly, our results indicate that TGF-β1-stimulated phosphorylation of ERK 1/2, AKT, MLC and p38 were all significantly inhibited by PFD in both CT-and DD-derived fibroblasts. PFD treatment alone also significantly inhibited the basal phosphorylation levels of AKT in both CT- and DD-derived fibroblasts. An inhibition in the basal phosphorylation levels in the other molecules, ERK1/2, MLC, and p38 was noted although a statistical significance was not achieved.

ERK1/2 belongs to the mitogen-activated protein kinase superfamily and mediates cell proliferation, migration, and apoptosis [36]. p38 MAPKs normally respond to environmental stress and inflammatory cytokines and mediate cell differentiation and apoptosis [37]. Krause et al. (2011) have previously reported the increased basal expression of phosphorylated ERK1/2 MAP kinase levels in Dupuytren’s fibroblasts [14]. Further authors also reported that co-treatment with MAPKK1 inhibitor PD98059 and TGF-β type I receptor kinase inhibitor SB-431542 abrogated proliferation and contractile efficiency of Dupuytren’s fibroblasts. We observe here that PFD is able to accomplish a similar pharmacologic inhibition of ERK 1/2 inhibitors.

Akt is a downstream protein kinase of PI3K, and it is activated by phosphorylation of IP3K. The activated Akt kinase phosphorylates a number of key substrates involved in the stimulation of intermediary metabolism and promotion of cell survival, proliferation, and growth [38]. Similar to Akt kinase activated p38 has numerous possible downstream targets driving cell proliferation and ECM deposition [39, 40]. Earlier studies by Ratkaj et al. (2012) report higher levels of phosphorylated p38 and Akt kinases along with typical myofibroblast markers, α-SMA, and palladin in DD fibroblasts. Decreasing p-p38 and Akt levels using the inhibitor SB203580 reduced DD fibroblasts’ proliferative and differentiation potential to myofibroblasts [41]. In lieu of the findings of Ratkaj et al. (2012) [41] we show that basal phosphorylation levels of Akt was inhibited by PFD (trending towards statistical significance) in DD-derived fibroblasts. However, a significant decrease in basal phosphorylation levels of p38 by addition of PFD was noted in DD-derived fibroblasts. Our results further show that TGF- β1-induced phosphorylation of Akt and p38 was significantly inhibited by PFD in DD-cells again reiterating the potential of PFD in targeting molecules that mediate cell survival, proliferation, and growth.

Phosphorylation of myosin light chain (MLC) is associated with actin-myosin interaction to form stress fibers and contractile rings, facilitating cell contraction and motility [42]. We observed increased basal phosphorylation levels of MLC in DD fibroblasts significantly more than that was seen in the fibroblasts derived from CT (Fig. 4). In our earlier studies, we observed increased cell contractile property at the basal level in DD- derived fibroblasts versus CT-derived fibroblasts [21], which may be partly due to the elevated phosphorylation of MLC. Addition of PFD did not inhibit the basal phosphorylation levels of MLC in both CT-and DD-derived fibroblasts but inhibited the basal contractile property of both CT- and DD-derived fibroblasts [21] indicating that other factors may also play a role in the contractile machinery of cells.

Overall, results from our previous and present studies demonstrate that PFD may serve as a potential candidate to fight against Dupuytren’s fibrosis as it could attenuate both canonical and non-canonical profibrotic signaling molecules elicited by TGF-β1. Whereas other pharmacological inhibitors may only be of benefit in targeting a single specific signaling molecule in DD, using PFD may negate a requirement for multiple pharmacological inhibitors by targeting several signaling molecules simultaneously. Future studies will determine PFD’s actions on other growth factors, namely PDGF, VEGF, and FGF as each of these has been implicated in the progression and development of DD and collectively the pro-fibrotic effects elicited by all of these growth factors need to be targeted.

## Conclusions

Our previous published findings and current studies provide strong evidence of PFD’s potential in abrogating the TGF-β1 mediated profibrotic effects by inhibiting both SMAD- and non-SMAD induced signaling pathways. These findings strongly recommend PFD as a potential novel therapeutic agent for DD-fibrosis. Because PFD is already FDA-approved, we hope these findings will help illuminate the way towards a clinical trial to test its effects locally in the setting of actual in vivo disease.

## Abbreviations

CT: Carpal tunnel
DD: Dupuytren’s disease
p38: phosphorylated p38 mitogen-activated protein kinase
p-ERK: phosphorylated extracellular signal- regulated kinase
PFD: Pirfenidone
p-MLC: phosphorylated-myosin light chain
TGF-β_1_: Transforming growth factor-beta
α – SMA: Alpha-smooth muscle actin
